# Chemokine (C-X-C) Ligand 12 Facilitates Trafficking of Donor Spermatogonial Stem Cells

**DOI:** 10.1155/2016/5796305

**Published:** 2016-01-21

**Authors:** Zhiyv Niu, Shaun M. Goodyear, Mary R. Avarbock, Ralph L. Brinster

**Affiliations:** ^1^Department of Animal Biology, School of Veterinary Medicine, University of Pennsylvania, Philadelphia, PA, USA; ^2^Department of Medical Genetics, Mayo Clinic, Rochester, MN, USA

## Abstract

The chemokine (C-X-C) receptor type 4 (CXCR4) is an early marker of primordial germ cells (PGCs) essential for their migration and colonization of the gonads. In spermatogonial stem cells (SSCs), the expression of CXCR4 is promoted by the self-renewal factor, glial cell line-derived neurotrophic factor (GDNF). Here, we demonstrate an important role of CXCR4 during donor mouse SSCs reoccupation of the endogenous niche in recipient testis. Silencing of CXCR4 expression in mouse SSCs dramatically reduced the number of donor stem cell-derived colonies, whereas colony morphology and spermatogenesis were comparable to controls. Inhibition of CXCR4 signaling using a small molecule inhibitor (AMD3100) during the critical window of homing also significantly lowered the efficiency of donor-derived SSCs to establish spermatogenic colonies in recipient mice; however, the self-renewal of SSCs was not affected by exposure to AMD3100. Rather,* in vitro* migration assays demonstrate the influence of CXCR4-CXCL12 signaling in promoting germ cell migration. Together, these studies suggest that CXCR4-CXCL12 signaling functions to promote homing of SSCs towards the stem cell niche and plays a critical role in reestablishing spermatogenesis.

## 1. Introduction

The spermatogonial stem cell (SSC) is the foundation of an elegant system for the transmission of genetic material, as functional differentiation produces an exponential number of sperm carrying the haploid genome [1, 2]. Identifying the intrinsic and extrinsic factors regulating SSCs and their niche is important to understanding mechanisms regulating SSC self-renewal and differentiation and offers significant translational benefits towards treating male infertility, especially in regard to pediatric oncology where there is growing concern over the gonadotoxic side effects of radiation and chemotherapy on the fertility of prepubertal boys [1–3].

While considerable effort has been made to understand the SSC niche in the testis, there is still much that remains unknown. The spatial localization of SSCs within the testis niche is in part supported by Sertoli, peritubular myoid, and Leydig cells, as well as microvasculature, which all work in concert to produce soluble factors and extracellular matrix components that provide cues for self-renewal and differentiation [2, 4]. One example is Sertoli cell and peritubular myoid cell production of the soluble ligand, glial cell line-derived neurotropic factor (GDNF), which is a crucial factor regulating SSC self-renewal [5–8]. GDNF elicits its effects via the GDNF family receptor *α*1 (GFR*α*1) and regulates several downstream factors, including ets variant 5, B-cell CLL/lymphoma 6 member B, LIM homeobox 1, and microRNA-21; all of which are important in mediating SSC homeostasis [9, 10]. In addition to GDNF, several other factors including the chemokine (C-X-C motif) ligand 12 (CXCL12, also referred to as SDF-1) are shown to aid in maintaining the SSC niche [11–13].

CXCL12 is the cognate ligand for the chemokine (C-X-C) receptor type 4 (CXCR4), a G protein-coupled receptor (GPCR) [14]. Studies show that the CXCR4-CXCL12 signaling axis is important in promoting chemotaxis in a variety of cell types, including the recruitment and retention of bone marrow and germline stem cells to their respective niches [15, 16]. Notably, CXCL12 concentration gradients produced by the mesenchyme of the genital ridge recruit CXCR4 expressing primordial germ cells to colonize the gonads [16]. The importance of CXCR4-CXCL12 signaling in maintaining SSCs was unclear until recent studies showed that perturbation of CXCR4-CXCL12 signaling in the testis of recipient mice using a small molecule inhibitor, AMD3100, drastically reduced donor-germ cell colonization [12]. Further evaluation of this pathway has revealed that silencing CXCR4 expression in SSCs, or depletion of the CXCR4 expressing SSC population, greatly reduces the colonizing potential of donor-derived germ cells in recipient testes [11, 12]. In this study we elaborate on the contribution of CXCR4-CXCL12 signaling and further examine its ability to promote effective homing of SSCs to their stem niche.

## 2. Methods

### 2.1. Germ Cell Isolation and Culture

Mouse SSC-enriched germ cell cultures were established from C57BL/6 (Stock no. 000664; The Jackson Laboratory) or ROSA26 (Stock no. 002073; The Jackson Laboratory) mice as previously described [17]. In brief, the testes from postnatal day 6–8 pups were collected and digested with 0.25% trypsin-ethylenediaminetetra-acetic acid (EDTA; Life Technologies) containing 7 mg/mL of DNase I (Sigma), followed by removal of nonviable cells and debris by centrifugation on a 30% Percoll gradient (600 ×g, 4°C, 7 min). A microbead-conjugated antibody against THY1.2 (Miltenyi Biotec) was used to enrich the SSC fraction and these cells were cultured on mitotically inactivated SIM mouse embryo-derived thioguanine- and ouabain-resistant (STO) feeders at a density of 0.5–1.0 × 10^5^ cells/well. The SSC-enriched germ cell cultures were maintained in a previously described chemically defined, serum-free minimal essential medium alpha (MEM*α*) medium (mSFM) [17]. The medium was replaced every 2-3 days and germ cell cultures were passaged approximately every 7 days. For* in vitro* quantification of SSCs, germ cell cultures were digested using trypsin-EDTA and the total number of cells (STO feeders plus germ cell clumps) was counted and subtracted from the number of mitotically inactive STO feeders initially seeded (i.e. 1.5 × 10^5^ cells). The Institutional Animal Care and Use Committee of the University of Pennsylvania approved all animal protocols (Protocol #702099).

### 2.2. Immunohistochemistry and Immunocytochemistry

Testes from adult (6-month old) mice were isolated and fixed in 4% paraformaldehyde. The testes were processed by the Histology and Gene Expression Core at University of Pennsylvania. Tissue sections were deparaffinized, hydrated, and subject to sodium citrate antigen retrieval. Tissue sections were blocked using 10% normal goat serum followed by 1 hr incubation at room temperature with anti-CXCR4 antibody (BD Bioscience; CD184, clone 2ab11) or a goat immunoglobulin (IgG) antibody. Samples were washed in PBS and incubated for 20 min with biotin-labeled secondary antibody at room temperature. Sections were washed and incubated with streptavidin-conjugated HRP and then developed using the HistoStain SP substrate kit (Life Technologies).

To examine protein expression in SSC-enriched germ cell cultures, cells were fixed with 4% paraformaldehyde for 20 min and permeabilized with 0.1% Triton X-100 in Dulbecco's PBS for 60 min at room temperature. After incubating with 20% normal donkey or goat serum for 60 min to avoid nonspecific interaction with antibodies, the cells were labeled overnight at 4°C with anti-CXCR4 antibody. The following day, cells were incubated with Alexa Fluor 488-conjugated donkey anti-rat IgG secondary antibodies for 5 hr at 4°C. Nuclei were labeled with 4′,6-diamidino-2-phenylindole (DAPI). The stained cells were analyzed using a Leitz Dialux 20 microscope (Leica Microsystems), and images were obtained with a Spot Insight 2MP Firewire Color Mosaic Digital Camera (Diagnostic Instruments).

### 2.3. Gene Expression Analysis

Total RNA (1 *μ*g) from transfected germ cell cultures or testis was isolated using Trizol and processed for first-strand cDNA synthesis, using TaqMan® Assay Kit (Life Technologies) according to manufacturer directions. Gene expression was assessed by qRT-PCR using commercially available TaqMan Gene Expression primers for mouse GATA4, GDNF, CSF-1, CXCL12, and GAPDH. Gene amplification was quantified using the ABI7500 detection system and accompanying software (Life Technologies). Relative gene expression was quantified using the 2^ΔΔCt^ method. All data are representative of at least three biological replicates with statistical significance between samples calculated using one-way ANOVA (*p* < 0.05).

### 2.4. Germ Cell Proliferation and Viability

To test whether CXCR4/CXCL12 regulates in SSC expansion, equal concentration of SSC germ cell culture was plated on STO feeder and treated with the specific CXCR4 inhibitor, AMD3100 (1.25 *μ*M; Sigma), or recombinant CXCL12 (10 ng/mL; R&D Systems), or both. Germ cells were cultured for 7 days after which they were gently dissociated from STO feeders and counted using a hemocytometer. In a repeat experiment, cell viability was assessed using the ATPlite assay kit per manufacturer (Perkin Elmer). In brief, 1 × 10^4^ germ cells were plated onto 96-well plates in defined culture media and treated with AMD3100, or the AKT inhibitor, LY29004 (33 *μ*M). At day 4 and day 6 after treatment, cell viability was assessed by quantifying ATP-dependent luciferase activity.

### 2.5. shRNA Lentiviral Transduction

Lentiviral-mediated short hairpin RNA (shRNA) was used in the stable repression of CXCR4 expression in SSC-enriched germ cell cultures. To create lentiviral particles, commercially obtained pLKO.1-puro vectors, containing* Cxcr4* shRNA or scrambled control shRNA (Sigma-Aldrich, USA), were cotransfected with envelope (pMD2.G) and packaging (psPAX2) plasmids into HEK293 packaging cells (ATCC) using Lipofectamine 2000 (Life Technologies). A single cell suspension of cultured germ cells was incubated with* Cxcr4* shRNA virus or control virus at a multiplicity of infection (MOI) of 5 in the presence of 8 *μ*g/mL polybrene for 18 hours. Cells were allowed to recover for 24 hours following which puromycin (1 *μ*g/mL) was used for 72 hours to select cells that stably incorporated the vector.

### 2.6. Germ Cell Transplantation

As previously described, SSC-enriched germ cell cultures (1 × 10^4^ cells/testis) were transplanted into the testis of adult, Busulfan-treated (60 mg/kg) 129/SvCP × C57BL/6 recipient mice (stock no. 101043; The Jackson Laboratory) [18]. A total of 3 recipient mice (i.e., *n* = 6 testes) were used per treatment group. The SSC-enriched germ cell cultures derived from ROSA26 mice were used to enable detection of *β*-galactosidase activity in recipient testis. Two months after transplantation the testes were harvested and analyzed for donor cell-derived colonies of spermatogenesis by staining with 5-bromo-4-chloro-3-indolyl-beta-D-galacto-pyranoside (X-gal; Thermo Fisher Scientific). All X-gal stained colonies that formed within tubules from recipient testis were counted. SSC colony formation was calculated according to the established formula: SSC number per 10^5^ THY1^+^ cells cultured = (number of donor-derived colonies of spermatogenesis) × (10^5^ total cells harvested/10^5^ cells transplanted) × (1/10^5^ THY1^+^ cells originally cultured).

*In vivo* investigation of CXCR4-CXCL12 signaling in SSC transplantation assays required repeated injection of AMD3100 into mice. This is due to the short half-life of AMD3100, which is rapidly metabolized and cleared out of the body [19], thus providing an opportunity for donor SSCs to migrate to their stem cell niche using CXCL12/CXCR4 signaling.

### 2.7. Germ Cell Migration Assay

Culture mouse germ cells were harvested and depleted of feeder cells by two rounds of preplating. 10^5^ mouse germ cells in single cell suspension were then placed in the upper chamber of a transwell insert containing a polycarbonate membrane with 5 *μ*m pores (Cell Biolabs Inc.). The transwell insert was placed into a well of a 12-well plate containing serum-free medium with or without recombinant CXCL12/SDF-1 (R&D systems; 10 ng/mL) and AMD3100 (Sigma Aldrich, 1.25 *μ*M) [19]. Cultures were incubated for 24 hrs following which the media containing migrated germ cells were collected and cells stained with cell stain solution (Cell Biolabs Inc.). The absorbance of invading germ cells was quantified at OD at 562 nm. Cell stain solution in blank wells containing only medium was used as the reference solution. The higher the number of invading germ cells in the bottom well is, the greater the absorbance quantified is.

## 3. Results

### 3.1. Expression of CXCR4 and CXCL12 in the Rodent Testes

**Figure 1:**
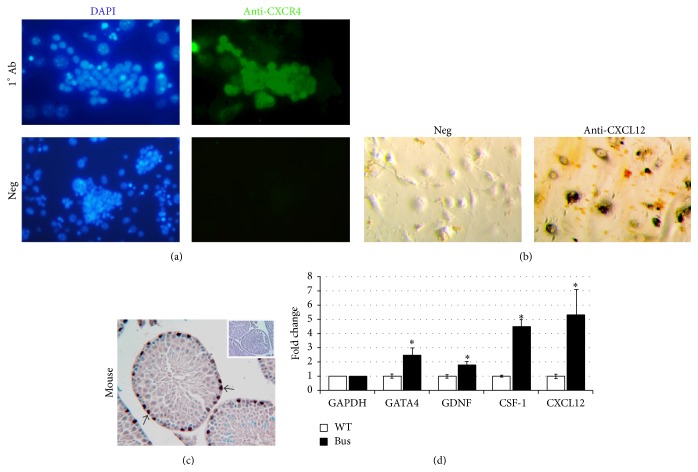
*In vitro* and* in vivo* expression analysis of CXCR4/CXCL12 in rodent testis cells. (a) Expression of CXCR4 membrane receptor in SSC-enriched cultured germ cells was determined by immunofluorescence using FITC-labeled anti-CXCR4 antibody (green), with the cell nucleus labeled by DAPI (blue). (b) Immunohistochemistry staining of CXCL12 protein in the STO feeder cells. (c) Immunohistochemistry staining of CXCR4 in mouse testis. Arrows show CXCR4 staining of germ cells on the basement membrane of seminiferous tubule. (d) The fold change in gene expression of GATA4, GDNF, CSF-1, and CXCL12 in the testes of untreated and Busulfan-treated recipient mice was verified by qRT-PCR. Changes in gene expression are normalized to GAPDH. Significance (denoted as *∗*) was calculated using Student's *t*-test (with *p* < 0.05, *n* = 3). Data are means ± SEM.

Prior reports show that the transcript levels of CXCR4 are markedly elevated in mouse THY1^+^ enriched SSCs compared to somatic testis cells [10]. SSC-enriched germ cell cultures, but not STO feeder cells, showed strong expression of CXCR4 (Figure 1(a)). Conversely, the CXCL12 ligand was intensely expressed by the STO cell feeder layer (Figure 1(b)). Immunohistochemical (IHC) evaluation of mouse testis showed that CXCR4 expression was localized to spermatogonia residing on the basement membrane of the seminiferous tubules (Figure 1(c)). Next the gene expression of the Sertoli cell marker, GATA4, was evaluated in relation to GDNF and known GDNF-regulated genes, CSF-1 and CXCL12. Compared to testes from untreated mice, an increase in the expression of GATA4 was correlated with elevations in the expression of GDNF, CSF-1, and CXCL12 in the testis of Busulfan-treated mice (Figure 1(d)).

### 3.2. Impact of CXCR4-CXCL12 Signaling on SSC Homeostasis

**Figure 2:**
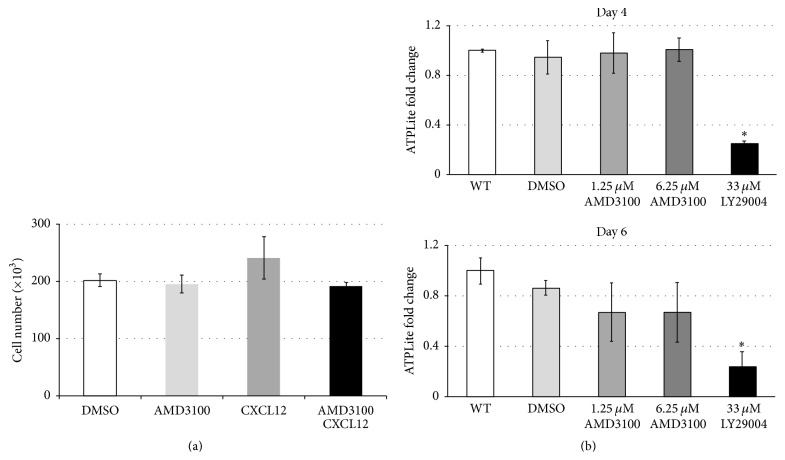
Effects of CXCR4 inhibitor AMD3100 on SSC-enriched THY1^+^ mouse germ cells. (a) An equal number of SSC-enriched cultured germ cells (1 × 10^5^ cells/well) were cultured on a layer of STO feeder cells and were treated with the vehicle (DMSO) or treated with AMD3100 (1.25 *μ*M), CXCL12 (10 ng/mL), or the combination of both CXCL12 and AMD3100. After 7 days the total number of cultured germ cells was quantified by cell counting. Values are representative of three independent experiments, and significance was calculated using one-way ANOVA. Data are means ± SEM. (b) Impact of AMD3100 on germ cell viability. SSC-enriched cultured germ cells were seeded on feeder-free, laminin-coated 96-well plates at a density of 1 × 10^4^ cells/well. Cells were either untreated (WT) or treated with DMSO control, AMD3100 (1.25 *μ*M and 6.25 *μ*M), or AKT inhibitor, LY29004 (33 *μ*M). Cell viability was assessed at days 4 and 6 following treatment using the ATPLite assay. Data are means ± SEM, where significance (denoted as *∗*) was calculated using Student's *t*-test (*p* < 0.05, *n* = 4).

To determine the influence of attenuating CXCR4-CXCL12 signaling on SSC containing germ cell cultures, the cultures were treated with the small molecule inhibitor, AMD3100 [12]. Treatment with AMD3100 had little to no effect on the proliferation or viability of THY1^+^ germ cell cultures (Figures 2(a) and 2(b)), whereas treatment with the PI3K-Akt inhibitor, LY29004, dramatically reduced the viability of SSC-enriched germ cell cultures (Figure 2(b)).

**Figure 3:**
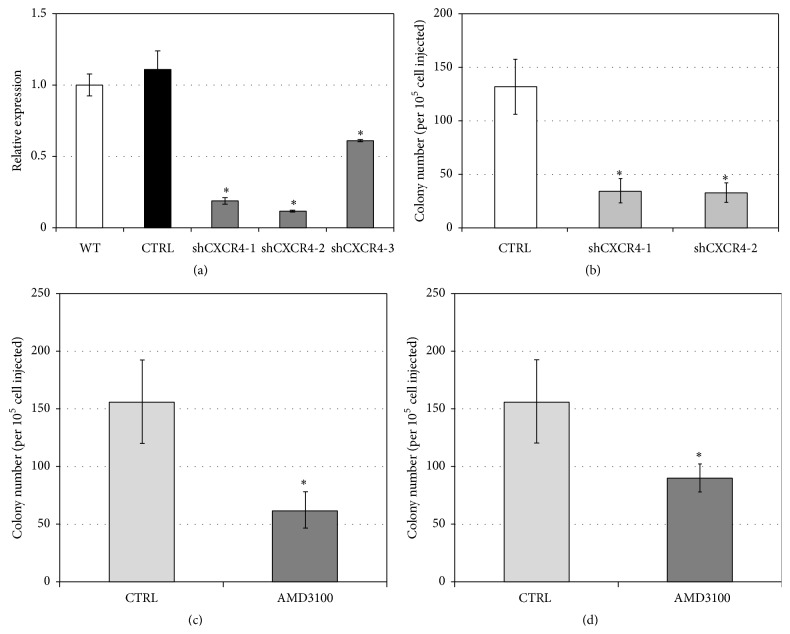
Inhibition of CXCR4 expression or function reduces stem cell activity of SSC-enriched germ cells. (a) qRT-PCR examination of shRNA-mediated knockdown of CXCR4 gene expression. SSC-enriched germ cell cultures were transduced with lentivirus containing a scrambled control shRNA vector or with one of three CXCR4-targeting shRNA clones (shCXCR4-1, shCXCR4-2, and shCXCR4-3). The transcript levels of CXCR4 were normalized to GAPDH gene expression and compared to untreated controls using 2^−ΔΔCT^. Significance was calculated using a Student's *t*-test (*p* < 0.05; *n* = 3). Data are means ± SEM. (b) Colony forming efficiency of CXCR4-deficient SSC-enriched germ cell cultures. CXCR4 shRNA lentiviral transduced cultures of SSC-enriched germ cells derived from Rosa-lacZ mice were transplanted at 1 × 10^4^ cells/testis into the testes of Busulfan-treated recipient mice. Donor-derived spermatogenesis was quantified by staining recipient testes with X-gal 2 months after transplantation. Significance was calculated using a Student's *t*-test (*p* < 0.05; *n* = 6 testes per treatment group). Data are means ± SEM. (c) AMD3100 disrupts SSC colony formation. Donor Rosa-lacZ SSC-enriched germ cells were treated with AMD3100 (5 *μ*g/testis) followed by transplantation into recipient testis (data are means ± SEM, *p* = 0.022, *n* = 4 testes per treatment group). (d) Alternatively, recipient mice bearing Rosa-lacZ SSC-enriched transplanted germ cells were repeatedly administered AMD3100 (5 *μ*g/testis) via the intraperitoneal (IP) route at 12 hr intervals for 2 days following transplantation. Donor-derived colonies were detected using X-gal and counted 2 months after transplantation. Data are means ± SEM, where significance was assessed using Student's *t*-test (*p* = 0.058, *n* = 2 testes per treatment group).

To further investigate CXCR4-CXCL12 signaling, CXCR4 expression was stably silenced in SSC-enriched germ cell cultures using commercially obtained shRNA lentiviral vectors. Initial screenings evaluated three CXCR4 shRNAs (shCXCR4-1, shCXCR4-2, and shCXCR4-3) and found that shCXCR4-1 and shCXCR4-2 provided the highest efficiency of gene targeting, reducing CXCR4 expression by ~80% compared to untreated germ cells or germ cells transduced with scrambled control shRNA (Figure 3(a)). Transplantation of the SSC-enriched germ cell cultures into the testes of recipient mice revealed that shRNA-mediated silencing of CXCR4 significantly reduced the number of spermatogenic colonies compared to control donor cells expressing scrambled shRNA (shCXCR4-1 = 34.3 ± 11.1 and CXCR4-2 = 32.9 ± 9.1 versus control = 131.4 ± 25.9; mean ± SEM; *p* = 0.02, *n* = 3) (Figure 3(b)). Next we investigated the importance of CXCR4-CXCL12 signaling in establishing spermatogenesis. Compared to control germ cell culture transplants, germ cell cultures transplanted into recipient testis along with AMD3100 showed a significant reduction in donor-derived spermatogenic colonies (156.0 ± 36.4 versus 62.1 ± 15.6 colonies/10^5^ cells injected; mean ± SEM; Student's *t*-test, *p* = 0.022, *n* = 4) (Figure 3(c)). In a similar experiment, recipient mice were repeatedly administered AMD3100 for 2 days following transplantation of donor germ cell cultures. Compared to untreated recipient controls, the repeated administration of AMD3100 dramatically reduced the number of donor-derived spermatogenic colonies, though this finding was nonsignificant (156.4 ± 36.15 versus 89.9 ± 12.0, colonies/10^5^ cells injected; mean ± SEM; Student's *t*-test, *p* = 0.058, *n* = 4 replicates) (Figure 3(d)). These results are in agreement with previous reports and highlight the importance of CXCR4-CXCL12 signaling axis in facilitating recolonization of the testis [11, 12].

### 3.3. CXCL12 Promotes Germ Cell Migration

**Figure 4:**
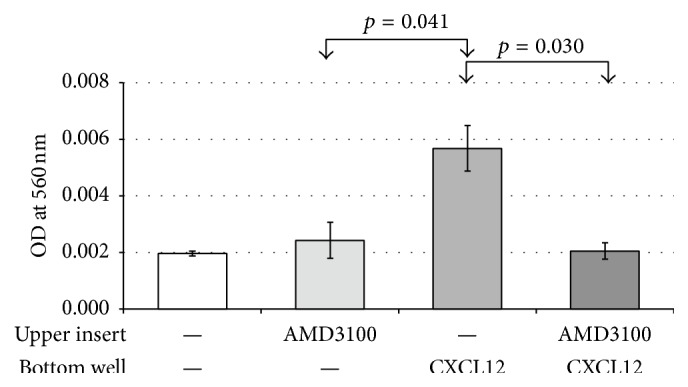
CXCL12/CXCR4 pathway promotes germ cell migration. SSC-enriched germ cells seeded in upper transwell in the presence or absence of AMD3100 (1.25 *μ*M) were exposed to CXCL12 (10 ng/mL) in the lower well. Measurement of the absorbance (562 nm) in the bottom well was used to assess the active number of germ cells that migrated from upper to lower wells. Data are means ± SEM, where significance was assessed using a Student's *t*-test (*p* < 0.05, *n* = 3 experimental replicates).

Although perturbing CXCR4-CXCL12 signaling had minimal effect on* in vitro* germ cell proliferation, the inhibition of the CXCR4-CXCL12 signaling significantly reduced the ability of donor-derived SSCs to reestablish spermatogenesis in recipient testis. Along with recent reports, our findings suggest that CXCR4-CXCL12 signaling has an important role in the homing of SSCs to the stem cell niche [11, 12]. To test this concept* in vitro*, we established a modified Boyden-chamber assay in which germ cell cultures were plated on a polycarbonate membrane insert with a pore size that is 5 *μ*m smaller than the diameter of mouse SSCs; therefore, cells located on the bottom well were representative of active cell migration. SSC-enriched germ cell cultures were treated with or without AMD3100, and the transwell insert was incubated in serum-free medium in the presence or absence of CXCL12 (10 ng/mL). After 24 hrs, the number of migrating germ cells was 2.89-fold higher in response to CXCL12 ligand compared to untreated controls (Figure 4). Conversely, inhibition of CXCR4-CXCL12 signaling using AMD3100 lowered the number of migrating germ cells to baseline levels (Figure 4). These results strongly suggest that CXCR4 regulates coordinated motility of undifferentiated germ cells in a CXCL12 ligand-dependent manner.

## 4. Discussion

In this study, we confirm previous findings involving CXCR4-CXCL12 signaling and its ability to mediate SSC colonization of the testis [11, 12]. Compared to controls, reduced colony formation in both CXCR4 shRNA SSCs, as well as those treated with AMD3100, reduced the number of donor-derived colonies in recipient testis. Our* in vitro* migration assay showed that perturbing CXCL12/CXCR4 signaling does not alter SSC proliferation (i.e., self-renewal), but rather this inhibition in signaling reduces SSC ability to migrate. From this finding, we would interpret that the reduced number of donor-derived colonies in AMD3100 treated testis is the result of inhibiting CXCL12/CXCR4 signaling in donor SSCs, which prevents them from relocating to the stem cell niche.

The localization of SSCs within the seminiferous tubules is dynamic and stage-dependent [20, 21]. As such, the processes governing SSCs involve a complex system of soluble signaling factors and cell-cell interactions that enables spatial localization of SSCs and coordinates their ability to self-renew or differentiate [22]. Several cytokines produced by Sertoli cells are strongly implicated in contributing to this signaling, including, CCL9, CXCL5, and CXCL12 [11, 12, 23, 24]. Additionally, the transmigration of SSCs from the lumen of the seminiferous tubule to the SSC niche located on the basement membrane is also regulated by the adhesion molecule *β*1-integrin and small GTP-binding protein RAC1 (ras-related C3 botulinum toxin substrate 1) [25, 26]. Interestingly, as a member of the Rho family of GTPases, RAC1 has a role in CXCR4 mediated cellular motility of CD34^+^ hematopoietic stem cells [27]. Future studies are required to determine whether CXCR4 directed activation of RAC1, or another Rho family of GTPase, functions to mediate SSC transmigration to its respective niche within the basement membrane.

Unlike the Busulfan-treated testes that are devoid of endogenous SSCs, the untreated testes contain both somatic cells and germ cells. Thus, the elevated levels of GATA4, GDNF, CSF-1, and CXCL12 mRNA are likely reflective of the remaining somatic cells that constitute the tubules (e.g., Sertoli cells, peritubular myoid cells). While largely quiescent, SSCs will enter the cell cycle and increase proliferation at a higher frequency following the loss of germ cells following injury [28]. Fittingly, a recent study has shown that cisplatin-mediated injury of the mouse testis dramatically increases the production of GDNF [29]. Together with our findings, we would suggest that acute gonadotoxicity in Busulfan-treated mouse testes increases production of GDNF, CSF-1, and CXCL12 in response to an absence of germ cells to promote retention of SSCs to its niche on the basement membrane. Because SSCs are enriched for both CSFR1 and CXCR4 expression, it strongly suggests that the donor SSC is readily primed for activation by these ligands [6, 7, 12, 30, 31]. As a possible damage response, this mechanism would provide a homing signal for surviving endogenous SSCs to be maintained within the stem cell niche (i.e., SSC homing to the basement membrane mediated by CXCR4-CXCL12 signaling). Subsequently, the elevated production of GDNF and CSF-1, as well as other factors, would promote SSC survival and enhance self-renewal [6, 7, 26, 30]. Our finding of elevated transcript levels of GDNF, CSF-1, and CXCL12 in Busulfan-treated testis suggests that this may be the case; however, further studies are required to evaluate protein expression and cellular localization of ligands in somatic cells that constitute the seminiferous tubules. Likewise, additional experiments are needed to track the* in vivo* spatial of movement of the donor SSC.

In pediatric oncology there is growing concern regarding the gonadotoxic side effects of radiation and chemotherapy on the fertility of prepubertal boys [32–34]. As a result, there are an increasing number of institutions that are offering fertility preservation by cryopreserving testis biopsies for future autologous transplantation of the SSCs [35]. This approach holds great potential as a regenerative stem cell therapy to restore patient fertility but still faces several obstacles, including enrichment of patient SSCs and separation from possible contaminating cancer cells [32, 35]. Barring such obstacles, the possibility of autologous SSC transplantation in patients is close to being realized. In this report, the results of using a Busulfan mouse model suggest another important aspect for clinical consideration:* what changes in the expression of essential SSC niche factors occur in response to gonadotoxins or the absence of germ cells?* While biochemical evaluation of gonadotropins and androgen levels are essential in evaluating patient recovery from exposure to gonadotoxins, there is a clear lack of knowledge regarding how similar exposures affect the expression of SSC niche factors (e.g., GDNF, CSF-1, and CXCL12) [36]. Understanding such changes will improve the clinical success of patients regaining complete fertility following autologous SSC transplantation.

## 5. Conclusions

We show that the CXCR4-CXCL12 signaling axis functions to promote homing of SSCs to the stem cell niche and plays a critical role in reestablishing spermatogenesis. Moreover, we demonstrate that Busulfan treatment of recipient mice increases the basal levels of GDNF, CSF-1, and CXCL12 expression, suggesting that Busulfan-mediated gonadotoxicity alters the testis microenvironment. Additional studies using Busulfan-treated testis or W/Wv mouse testis will shed further light on other soluble factors and signaling events that would enable an appropriate response to facilitate recruitment of the SSCs to their niche.
